# Fusion peptide constructs from antigens of *M*. *tuberculosis* producing high T-cell mediated immune response

**DOI:** 10.1371/journal.pone.0271126

**Published:** 2022-09-29

**Authors:** Shaista Arif, Mohsina Akhter, Aasia Khaliq, Muhammad Waheed Akhtar

**Affiliations:** 1 School of Biological Sciences, University of the Punjab, Lahore, Pakistan; 2 Department of Biology, Lahore University of Management Sciences (LUMS), Lahore, Pakistan; Centenary Institute, AUSTRALIA

## Abstract

Non availability of effective anti-TB vaccine impedes TB control which remains a crucial global health issue. A fusion molecule based on immunogenic antigens specific to different growth phases of *Mycobacterium tuberculosis* can enhance T-cell responses required for developing a potent vaccine. In this study, six antigens including EspC, TB10.4, HspX, PPE57, CFP21 and Rv1352 were selected for constructing EspC-TB10.4 (bifu25), TnCFP21-Rv1352 (bifu29), HspX-EspC-TB10.4 (trifu37), HspX-TnCFP21-Rv1352 (trifu44) and HspX-EspC-TB10.4-PPE57 (tetrafu56) fusion proteins. Th1-cell epitopes of EspC, PPE57 and Rv1352 antigens were predicted for the first time using different *in silico* tools. The fusion molecule tetrafu56, which consisted of antigens from both the replicating and the dormant stages of *Mtb*, induced a release of 397 pg/mL of IFN-γ from PBMCs of the active TB patients. This response was comparable to the response obtained with cocktail of the component antigens (396 pg/mL) as well as to the total of the responses obtained separately for each of its component antigens (388 pg/mL). However, PBMCs from healthy samples in response to tetrafu56 showed IFN-γ release of only 26.0 pg/mL Thus a previous exposure of PBMCs to *Mtb* antigens in TB plasma samples resulted in 15-fold increase in IFN-γ response to tetrafu56 as compared to the PBMCs from the healthy controls. Hence, most of the T-cell epitopes of the individual antigens seem to be available for T-cell interactions in the form of the fusion. Further investigation in animal models should substantiate the immune efficacy of the fusion molecule. Thus, the fusion tetrafu56 seems to be a potential candidate for developing an effective multistage vaccine against TB.

## 1. Introduction

Tuberculosis (TB) is the leading cause of human deaths worldwide from a single infectious agent, which is *Mycobacterium tuberculosis (Mtb)*. It remains a great health menace to the world particularly with the emergence of multi and extensively drug resistant strains of *Mtb*. In 2019, 1.2 million deaths and 10 million new active TB cases were reported by WHO [1]. Globally, effective control of TB is based on early diagnosis, proper treatment and efficacious vaccine development. Protection against *Mtb* is based on the production of strong cell-mediated immune (CMI) responses involving the release of multiple cytokines like IFN-γ, TNF-α and IL-2 which actuate the invaded macrophages to eliminate the intracellular *Mtb* [2]. The production of IFN-γ by activated macrophages, natural killer cells, Th1 cells, and dendritic cells is predominantly related with protection against *Mtb* [3]. BCG, the only vaccine available for TB confers inconsistent protection against pulmonary TB in adults [4]. Therefore, there is a dire need to develop more effective subunit vaccines consisting of immunogenic antigens of *Mtb* to provide protection against *Mtb* [5].

Fusion molecules based on multiple *Mtb* antigens can induce strong protective CMI responses in genetically heterogeneous human populations [6]. A recent review has dealt with different approaches including the use of fusion antigens, for developing a TB vaccine [7]. A pre-exposure booster vaccine, made up of Rv1196 and Rv0125 showed 54% vaccine efficacy in the phase IIb trial for TB infected adults of Kenya, South Africa and Zambia [8]. Another fusion of Ag85B, ESAT-6 and Rv2600, designed for pre- and post-infection vaccination, is also under evaluation for prevention of TB recurrence [9]. Multistage subunit vaccine consisting of three early stage antigens Rv3620, Rv2608, Rv3619 and a latent stage antigen Rv1813 is under evaluation for its safety, tolerability and recurrence [10]. Although there are several reports with different levels of success, further work is required to achieve the targets of high efficacy and wide applicability of the vaccines.

The six T-cell epitopes containing *Mtb* antigens, which were reported specific to different stages of microbial growth and disease stages, included in this study were HspX, EspC, TB10.4, PPE57, CFP21 and Rv1352. EspC (*Rv3615c*) is a RD1-dependent secreted antigen of *Mtb* [11]. It can elicit significant T-cell responses in humans, inducing the production of Th1 cytokines like IL-2, IFN-γ and TNF-α, equivalent to the levels produced by ESAT-6 and CFP-10 [12,13]. A DNA vaccine (p846) containing EspC was also reported to induce specific Th1-type CD4^+^ response in mice [14]. PPE57 (*Rv3425*), a cell wall associated antigen belongs to PPE family and its locus is present in RD11 region of *Mtb* genome, which is absent in BCG strains [15,16]. The recombinant BCG containing Ag85B-PPE57 fusion antigen and PPE57 alone, generated higher levels of IFN-γ in antigen-activated T-cells [17]. TB10.4 (*Rv0288*) is an early secreted antigen which plays a key role in the intracellular survival of *Mtb* [18]. It contains multiple T-cell epitopes and induces an increased level of IFN-γ secretion than ESAT-6 in TB patients [19]. The multistage fusion-proteins Ag85B-TB10.4-Rv2660c, TB10.4-HspX and TB10.4-Ag85B also elicited strong host CMI responses [20–23].

HspX (*Rv2031c*) is a cytosolic protein with a chaperone-like activity and performs the protective role during intracellular stress [24]. It can generate both antibody and CMI responses in active and latent stages of *Mtb* infection [25]. A subunit vaccine consisting of Ag85B‐Mpt64_190–198_‐HspX fusion protein generated high levels of IFN-γ responses in mice [26]. Rv1352 is an uncharacterized outer cell membrane protein which showed a strong immune response [27]. CFP21 (Rv1984c) belongs to RD2 region of *Mtb* genome that has been reported to produce high levels of IFN-γ and IL-12 responses [28].

These six antigens and their fusion constructs EspC-TB10.4 (bifu25), TnCFP21-Rv1352 (bifu29), HspX-TnCFP21-Rv1352 (trifu44), HspX-EspC-TB10.4 (trifu37) and HspX-EspC-TB10.4-PPE57 (tetrafu56) were evaluated for their potential to induce T-cell specific IFN-γ response from human PBMCs. The fusion tetrafu56 generated 15-fold higher IFN-γ response from PBMCs of TB patients than the healthy individuals thus it seems to be a potential base molecule for developing a multistage subunit vaccine against TB.

## 2. Materials and methods

### 2.1 Prediction of epitopes and molecular modelling

Six immunogenic *Mtb* antigens EspC (*Rv3615*), HspX (*Rv2031c*), TB10.4 (*Rv0288*), PPE57 (*Rv3425*), CFP21 (*Rv1984*) and Rv1352 were selected for this study. Using immune epitope database (IEDB) (http://www.immuneepitope.org) [29], the T-cell epitopes of HspX, CFP21 and TB10.4 were retrieved. Since no T-cell epitope data was available for EspC, PPE57 and Rv1352, their potential Th1-cell epitopes were predicted for the first time by determining binding affinity of the antigens to MHC class II molecules through binding prediction tool using the consensus approach available on IEDB Analysis Resources (IEDB-AR) (http://tools.iedb.org/mhcii/) [30]. A panel of 15 HLA-DR, 6 HLA-DP and 6 HLA-DQ alleles were selected which included MHC class-II supertypes and the most common allelic variants based on their high allelic frequency present in Pakistani human population and most other ethnic groups [31]. All 15-mer peptides were selected for their strong binding affinity to HLA class II molecules with IC_50_ (inhibitory concentration) values of <50 nM. The peptide regions were further verified by NetMHCIIpan 4.0 predictor (https://services.healthtech.dtu.dk/service.php?NetMHCIIpan-4.0) based on artificial neural networks (ANN) method. The strong binding peptides with a rank value of 1% or less were selected as potential Th1-cell epitopes [32]. Furthermore, HLA-DR promiscuous peptides of EspC and PPE57 were confirmed using Propred web server (http://www.imtech.res.in/raghava/propred/) [33] at a default threshold value of 3.0. It is a web server using matrix-based algorithm for locating the promiscuous MHC class II binding regions of the antigenic proteins that can bind to 51 HLA-DR giving 95% population coverage. The peptides found to bind more than 50% of serologically defined HLA-DR molecules were predicted as promiscuous Th1-cell binders [34]. 3-dimensional structures of proteins were predicted using Raptor-X Server and validated through Verify 3D and Procheck as described previously [35,36]. Moreover, solvent accessibility analysis of the predicted models was determined by CPORT online web server (http://haddock.science.uu.nl/services/CPORT) [37]. The 3D structures were visualized using Pymol graphical program by Schrodinger.

### 2.2 Cloning and expression of the antigens and their fusions

**Fig 1:**
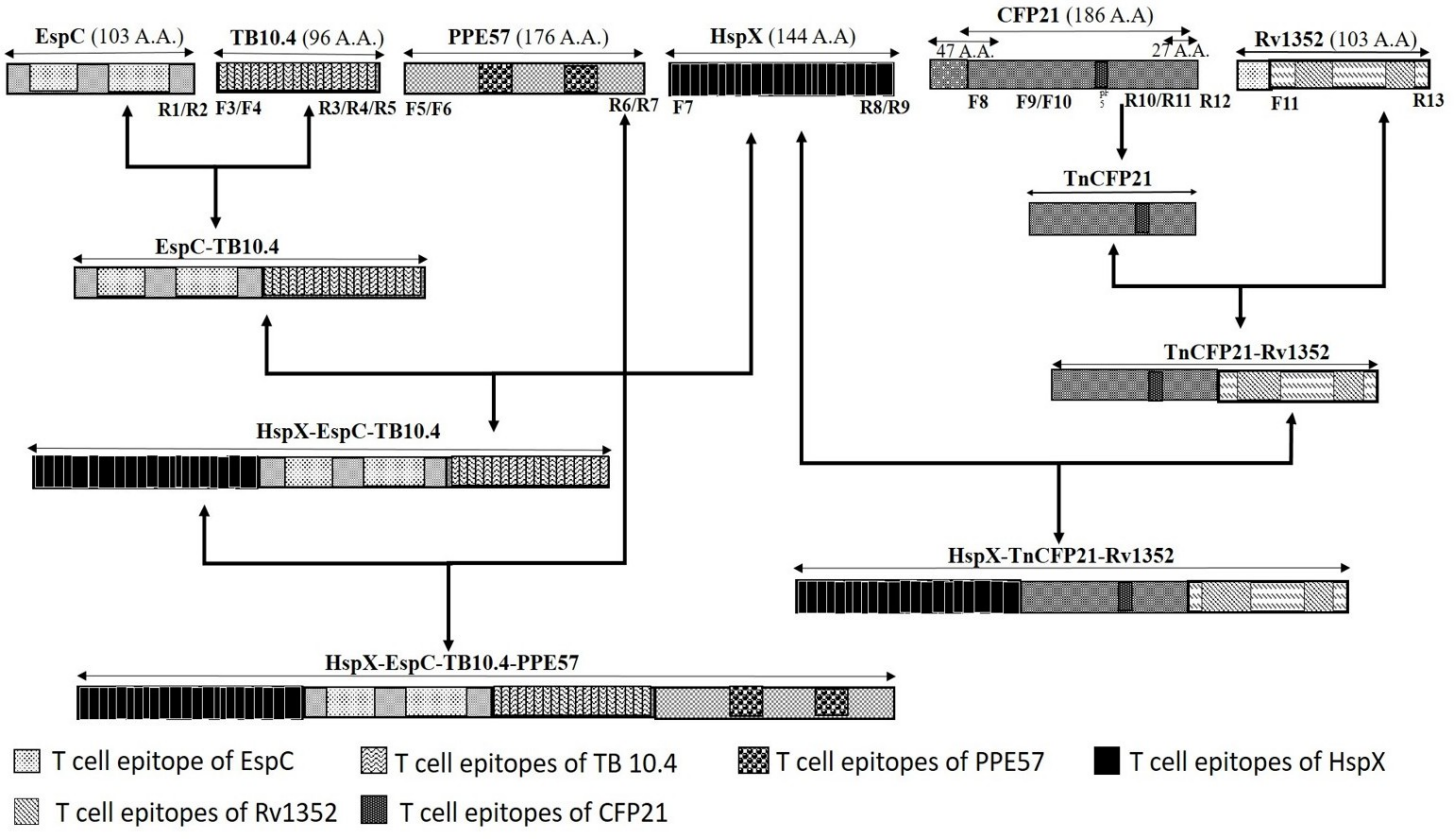
T-cell specific epitopes of the antigens used in this study as the scheme for construction of the fusion molecules.

For amplification of DNA sequence encoding *Mtb* antigens EspC (*Rv3615*), HspX (*Rv2031c*), TB10.4 (*Rv0288*), PPE57 (*Rv3425*), CFP21 (*Rv1984*) and Rv1352 genomic DNA of *Mtb H37Rv* was isolated and used as template [38]. The ORFs of *Rv3615c*, *Rv2031c*, *Rv0288*, *Rv3425*, *Rv1984c* and *Rv1352* encoding *EspC* (312bp), *HspX* (435bp), *TB10*.*4* (294bp), *PPE57* (531bp), *CFP21* (558bp) and *Rv1352* (312bp) antigens, respectively were PCR amplified using forward and reverse primers, shown in Table 1. To construct fusion proteins, genes encoding *HspX*, *EspC*, *TB10*.*4*, *PPE57*, *CFP21* and *Rv1352* antigens were amplified using primer sets F7-R9, F2-R2, F4-R4 & F4-R5, F6-R7, F9-R10 and F11-R13, respectively (Table 1). For developing multiepitope fusion constructs, the DNA fragments were cloned sequentially, using compatible restriction sites according to the scheme shown in Fig 1. The complete detail of the scheme used for the synthesis of fusion molecules can be followed from the information provided in Table 1 and Fig 1. Each of the fragment after amplification and restriction with the corresponding enzymes were gel purified [35,36]. The correct sequence and integration of the DNA inserts in all the recombinant plasmids were confirmed commercially (1^st^ Base company, Malaysia).

**Table 1:** Primers for PCR amplification with the restriction sites shown in bold.

Antigen	Primer	Sequence (5 ‘ 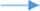 3’)	Restriction sites	Annealing temperature (°C)
**HspX**	F7	GCATTG **CATATG** GCCACCACCCTTCCCGTTC	*Nde*I	76. 3
	R8	ACGGACCCAGTGGTCAGTTGG	*None*	65.3
	R9	TCAAT **GGATCC** GGT GGC AGC GTT GGT GGA CC	*Bam*HI	77.6
**EspC**	F1	GGA**CATATG**ACGGAAAACTTGACCGTC	*Nde*I	68.2
	R1	TTA**AAGCTT**TCAGGTAAACAACCCGTCGATAG	*HindIII*	70.1
	F2	CTGGTA**GGATCC**ATGACGGAAAACTTGACCGTC	*Bam*HI	75.2
	R2	CATAT**GAATTC**GGTGGCGGCAGCGGTAAACAACCCGTC	*EcoR1*	79.4
**TB10.4**	F3	G**CATATG**TCGCAAATCATGTACAACTACC	*Nde*I	67.4
	R3	T**GAATTC**CTAGCCGCCCCATTTG	*EcoR1*	64.6
	F4	TCTGA **GAATTC** ATGTCGCAAATCATGTACAAC	*EcoR1*	68.7
	R4	TATAC**AAGCTT**CTAGCCGCCCCATTTGG	*HindIII*	70.1
	R5	TAT**AAGCTT**GGTAGCGCCGCCCCATTTG	*HindIII*	71.6
**PPE57**	F5	GTCTA**CATATG**CATCCAATGATACCAG	*Nde*I	65.3
	R6	GTAT**AAGCTT**CTACCCGCCCCTGTA	*HindIII*	67.4
	F6	ATCT**AAGCTT**ATGCATCCAATGATACCAGCGGAGT	*HindIII*	73.1
	R7	TTAT **GCGGCCGC** CTA CCC GCC CCT GTA	*Not1*	75. 9
**CFP21**	F8	**CATATG**GACCCGTGTTCGGAC	*Nde*I	63.2
	R12	T**AAGCTT**TCATCCGGCGTGATCG	*Hind*III	64.6
**Tn1CFP21**	F9	TA**GGATCC**GGCTCTCAGGCTTCTGGTC	*Bam*HI	72.2
	F10	**CATATG**CAGGCTTCTGGTCTTGG	*Nde*I	64.6
	R10	G**GAATTC**TTAAATATTGCCGCCTCC	*Eco*RI	64.1
	R11	ATATTA**GAATTC**GATGTTGCCGCCTCC	*Eco*RI	66.6
**Rv1352**	F11	TA**GAATTC**GCAATCACGCTCGC	*Eco*RI	62.1
	R13	A**CTCGAG**CTATGAGATCCGCATC	*Xho*I	64.6

For the expression of the recombinant proteins, competent cells of *E*. *coli* BL21-CodonPlus (DE3)-RIPL were transformed with the recombinant plasmids containing single and the fusion constructs. Cultivation of the cells was done in Luria Bertani (LB) medium at 37°C. The induction of bacterial culture was done with 0.5mM IPTG when OD_600_ reached 0.6–0.8 followed by further incubation for 5 hours. The harvested BL21cell were resuspended in 20mM Tris–Cl buffer (pH 8.0) containing 0.2M NaCl and 1mM PMSF and cell lysis was performed using Sonics Vibra-Cell VCX-500 Ultrasonic Processor. The cell lysates thus obtained were centrifuged at 7,000 rpm for 15 minutes to separate the soluble and the insoluble fractions. Solubility of the expressed protein was checked by the analysis of lysate supernatant and SDS PAGE analysis of lysate supernatants. Soluble proteins were purified through Ni-affinity chromatography. The inclusion bodies, if obtained, harvested through centrifugation at 3,000rpm for 20 minutes. After washing with 0.5% Triton X-100, the protein was solubilized in 20mM Tris–Cl (pH 8.0) containing 8M urea, 0.2M NaCl and 1mM PMSF. The solubilized proteins were purified through Ni^2^+-affinity chromatography [39]. Dialysis of the combined fractions containing the eluted proteins was done against 20mM Tris–Cl (pH 8.0). The molecular mass and purity of all the single and fusion proteins were assessed by 12% (v/v) SDS-PAGE. The percentage expression of all the recombinant proteins was analyzed by densitometric scanning of the SDS gel using Syngene gel documentation system (United Kingdom). Dye binding assay using BSA as a standard was performed to determine the protein concentrations [40].

### 2.3 Study subjects

The study was approved by the Institutional Review and ethics Board (IRB No. 00005281) of the School of Biological Sciences, University of the Punjab, Lahore, Pakistan (SBS/767/17) and the blood samples were collected during the period of Nov 2020–Dec 2020 and written consent was also obtained from all the study participants before collecting the blood samples. TB Patients were recruited according to the guidelines of World Health Organization (WHO) and National TB control Program for diagnosis and treatment of TB [41].

The healthy group consisted of 10 individuals (8 males and 2 females aged 22–36 years; mean age, 30 years). They had no clinical history of TB and were BCG vaccinated in childhood. The 21 active TB patients (17 males and 4 females aged 15 to 52 years; mean age, 32 years) were recruited from the outdoor patient department of Gulab Devi Chest Hospital, Lahore. All the TB patients were confirmed on the basis of clinical and radiological findings followed by direct AFB sputum smear microscopy and mycobacterial culturing. All the study subjects were HIV negative and they were not taking any immunosuppressive drugs. All the patient’s blood samples were drawn shortly after diagnosis and before the start of anti-TB treatment.

### 2.4 Isolation and *in vitro* stimulation of human PBMCs

PBMCs were isolated from heparinized peripheral blood after dilution with equal volume of 1x RPMI 1640 complete medium (Gibco Life Technologies) according to standard protocol with some modifications [42]. 5ml of the freshly diluted blood was layered on top of Ficoll‐Paque PLUS (GE Healthcare) and centrifuged at 2,000 rpm with no brakes for 10 minutes at 20°C. The mononuclear cell layer was isolated from underneath Ficoll-layer and washed twice with RPMI 1640 medium and finally resuspended in 0.5ml of the same medium supplemented with 2mM L-glutamine. The viable cell count was determined by treatment with 0.4% trypan blue dye (Sigma Aldrich) in a Countess^TM^ II FL Automated cell counter (Invitrogen). Freshly isolated PBMCs were resuspended in RPMI 1640 supplemented with 2mM L-glutamine, 10% heat inactivated fetal bovine serum (FBS) and 1% penicillin-streptomycin. 200 μl of cell suspension, after adjusting the cell count to 2.5 × 10^5^ were seeded in 96 well flat bottom culture plate (SPL Life sciences). Cells in each well were stimulated with 2.5μM of each of the single and the fusion constructs in duplicates. The cells were also cultured without antigens as unstimulated (negative) control and incubated at 37°C for 48 hours in a humidified air containing 5% CO_2_. After 48 hours of incubation, the culture supernatants were harvested and analyzed for the amount of human IFN-γ released.

### 2.5 IFN-γ assay

Amount of IFN-γ released in the PBMCs culture was determined using human IFN-γ ELISA kit (#EHIFNG; Invitrogen) according to the protocol recommended by the manufacturer. Recombinant human IFN-γ was used to plot a standard curve. 50 μl each of the biotinylated antibody reagent and the cell culture supernatant was added to each well of the pre-coated anti-human IFN-γ strips and incubated at 25°C for two hours. After washing thoroughly with the washing buffer, freshly prepared streptavidin-HRP solution was added to each well and further incubated at 25°C for 30 minutes. After washing, TMB substrate was added into each well and incubated at room temperature in dark for 30 minutes and reaction was terminated by adding stop solution. IFN-γ concentration (pg/mL) was determined by interpolating values from standard curve obtained with absorbance A_450nm_ minus A_550nm_.

### 2.6 Statistical analysis

The levels of IFN-γ release were statistically analyzed for significant differences between TB patients and the healthy subjects using non-parametric Mann-Whitney U test. All experiments were performed in duplicates for each sample. Results were presented as mean ± SD. The results were considered statistically significant for P <0.0001. Pearson’s rank correlation coefficient was used to analyze the correlation between IFN-γ levels of the fusion antigens and cocktails of the component antigens. Graphpad Prism (version 8.4.2) (GraphPad Software, San Diego, California, USA) and IBM SPSS statistics, version 23 (IBM Corp., Armonk, New York, USA) were used for the data analysis.

## 3. Results

### 3.1 Prediction of epitopes and molecular modelling

**Fig 2:**
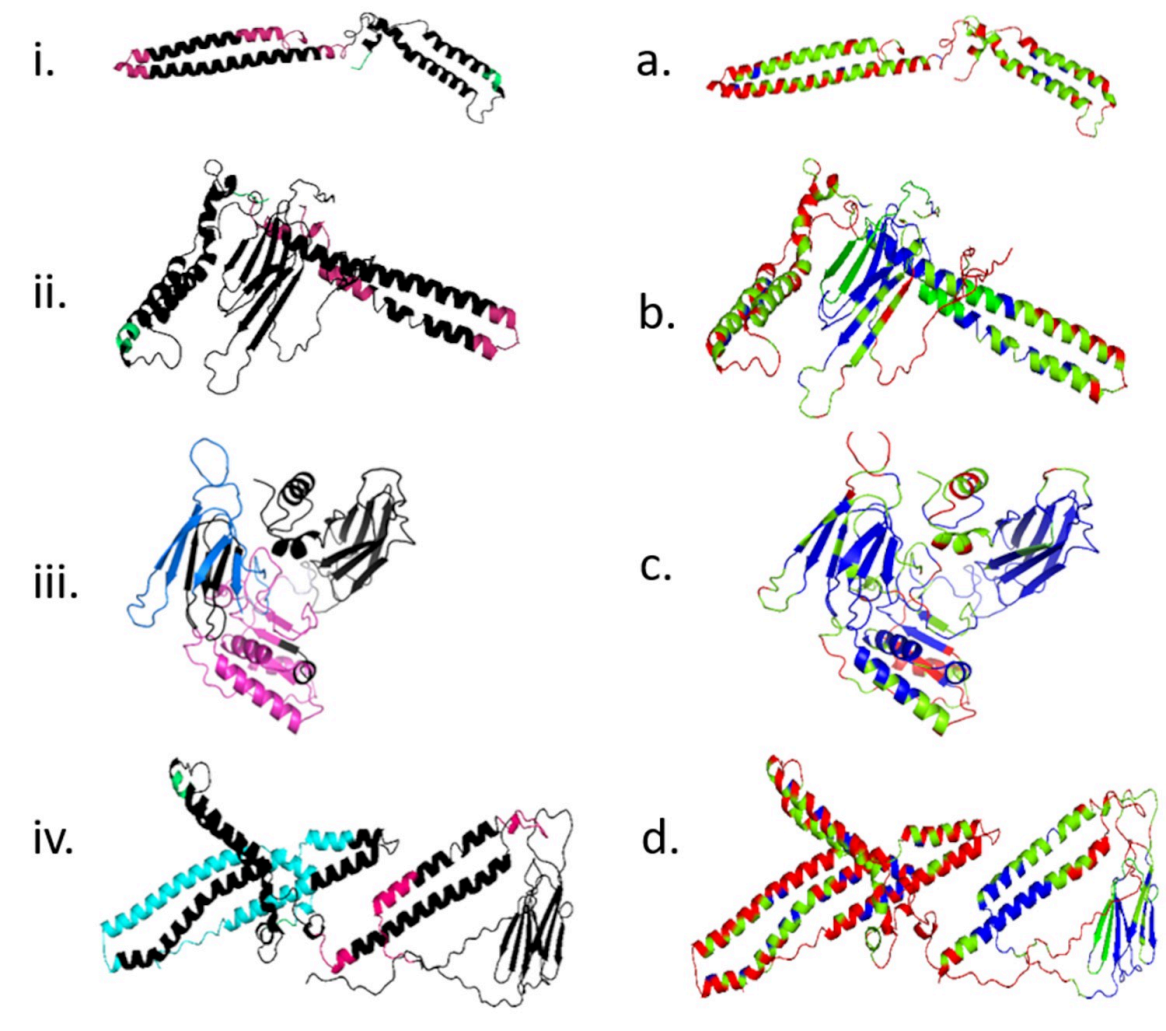
Structural analyses of the fusion antigens bifu25, trifu37, trifu44 and tetrafu56. T-cell epitopes in the 3D molecular structures (i-iv) are shown in black. CPORT analysis (a-d) shows the epitope regions as red, green or blue color, representing active, supporting or non-supporting residues, respectively for cellular interaction.

Th1-cell epitopes of EspC, PPE57 and Rv1352 antigens as predicted using IEDB MHC-II prediction software, showed two epitopes for each of these. The locations of the epitopes as confirmed by NetMHCIIpan 4.0 server are shown in Table 2. The already known Th1-cell epitopes of HspX, TB10.4 and CFP21, which numbered 39, 41, and 1, respectively, were retrieved from IEDB (http://www.immuneepitope.org). The predicted epitopes showing >50% binding affinity to HLA-DR molecules were represented as promiscuous peptides using Propred server (http://www.imtech.res.in/raghava/propred/). The single peptide of EspC: LRIAAKIYS with 84% and two peptides of PPE57: WVINGLANA and LQWLSKHSS with 73% and 61% binding affinity were further predicted as broadly recognized peptides. 3D structure and CPORT analyses of the four fusion molecules bifu25, trifu37, trifu44 and tetrafu56 are shown in Fig 2. Positions of the epitopes in the 3D structure (shown in black) and by CPORT analyses show that these are in a favorable position for interaction with T-cell receptors.

**Table 2:** Th1-cell epitopes predicted for the *Mtb* antigens.

Ser no.	Antigens	Length	Amino Acid sequence
1	PPE57	60–97	SDLLADAVERYLQWLSKHSSQLKHAAWVINGLANAYND
		115–142	RRRLIASNVAGVNTPAIADLAQYDQYR
2	Rv1352	18–35	GDGVFLVGTDIAPGTYRT
		84–99	IPPTVAAFQTHNCKL
3	EspC	19–38	HDNAAVDASSGVEAAAGLGES
		54–93	TLNVYLTAHNALGSSLHTAGVDLAKSLRIAAKIYSEADEA

### 3.2 Protein expression and purification

**Fig 3:**
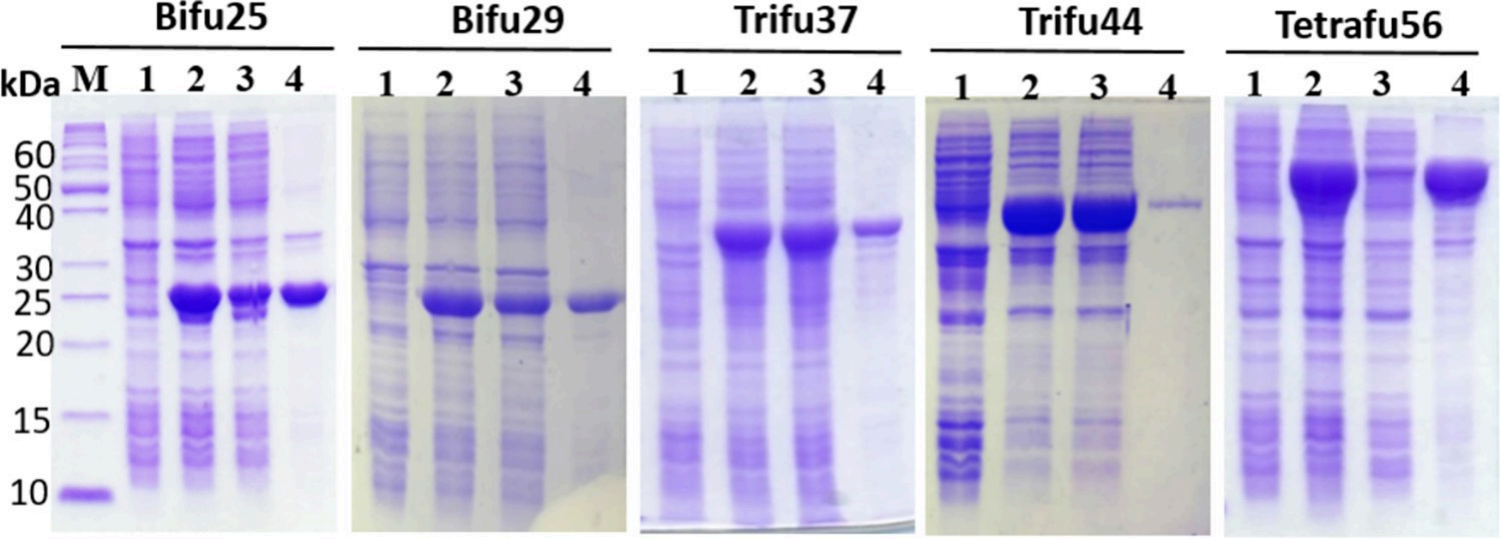
SDS-PAGE showing expression of bifu25 and bifu29 and the other fusion proteins. Lanes M: Protein markers; 1: Uninduced *E*. *coli* cells; 2: Lysate of the induced *E*. *coli* cells; 3: Soluble fraction of the cell lysate; 4: Insoluble fraction of the cell lysate.

After expression, molecular sizes and purification levels of all the recombinant proteins were analyzed on SDS-PAGE as shown previously [35,36]. Soluble expression of the trifusions produced by linking HspX to the N-termini of bifu25 and bifu29 fusions, not shown before, is presented in Fig 3.

### 3.3 IFN- γ release

**Fig 4:**
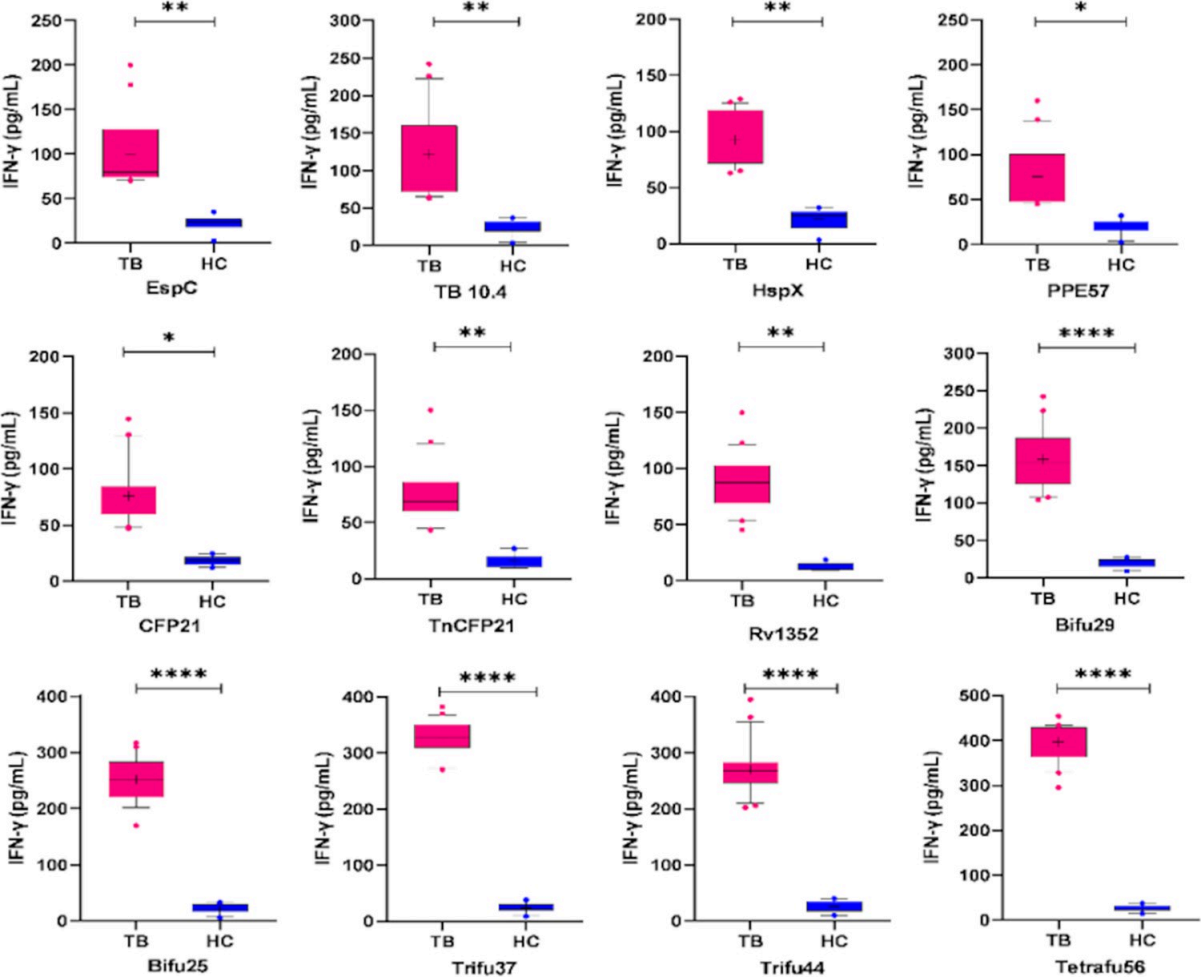
Box and whisker plots showing IFN-γ release against the single and their fusion antigens. * P >0.01, ** P *>*0.001, *** P *>*0.0001 and ****P < 0.0001. The vertical line inside each box represents median values. IFN-γ released by PBMCs from the active TB group are several-fold higher as compared to those from the healthy group (HC).

The amount of IFN-γ released in the cultures of PBMCs from the healthy and the TB patient group is represented in the form of box and whisker plots for each test antigen after subtracting the values of the unstimulated controls (Fig 4). The middle line in each box represented the median value for the IFN-γ release in response to the test antigens as mentioned in Table 3. The fusion antigen tetrafu56 showed the highest median IFN-γ release of 418 pg/mL with significant P value of <0.0001.

**Table 3:** Statistical data for the release of IFN-γ (pg/mL) by PBMCs from the TB and healthy subjects against the single antigens and their fusion constructs.

Antigens	Mean	Median	95% CI Lower-upper	Mean	Median	95% CI Lower-upper	P-value
	TB			HS			
EspC	100.3	80.0	82.7–117.8	21.7	23.8	14.7–28.7	0.0041
TB10.4	121.4	100.0	94.8–148.0	25.7	30.2	18.0–33.5	0.0054
HspX	92.1	83.0	81.6–102.7	22.1	25.2	15.3–28.9	0.0088
PPE57	75.4	60.0	59.1–91.6	20.6	22.7	14.7–26.7	0.0183
CFP21	75.8	63.4	63.2–88.3	18.2	18.5	15.4–21.2	0.0162
TnCFP21	76.0	68.7	63.7–88.2	16.4	16.8	11.9–21.0	0.0099
Rv1352	86.7	77.1	75.3–98.2	13.5	14.6	11.2–15.9	0.0043
Bifu25 (EspC-TB10.4)	252.1	251.0	234.3–269.9	21.0	20.0	15.8–26.8	<0.0001
Bifu29 (TnCFP21-Rv1352)	158.2	153.4	139.4–176.9	20.8	23.3	16.4–25.3	<0.0001
Trifu37 (HspX-EspC-TB10.4)	325.1	328.0	310.2–340.0	24.0	22.5	18.2–29.9	<0.0001
Trifu44 (HspX-TnCFP21-Rv1352)	270.4	267.6	249.7–291.1	25.8	26.4	18.2–33.5	<0.0001
Tetrafu56 (HspX-EspC-TB10.4-PPE57)	397.0	418.0	377.2–416.7	26.0	23.6	20.6–31.1	<0.0001

**Fig 5:**
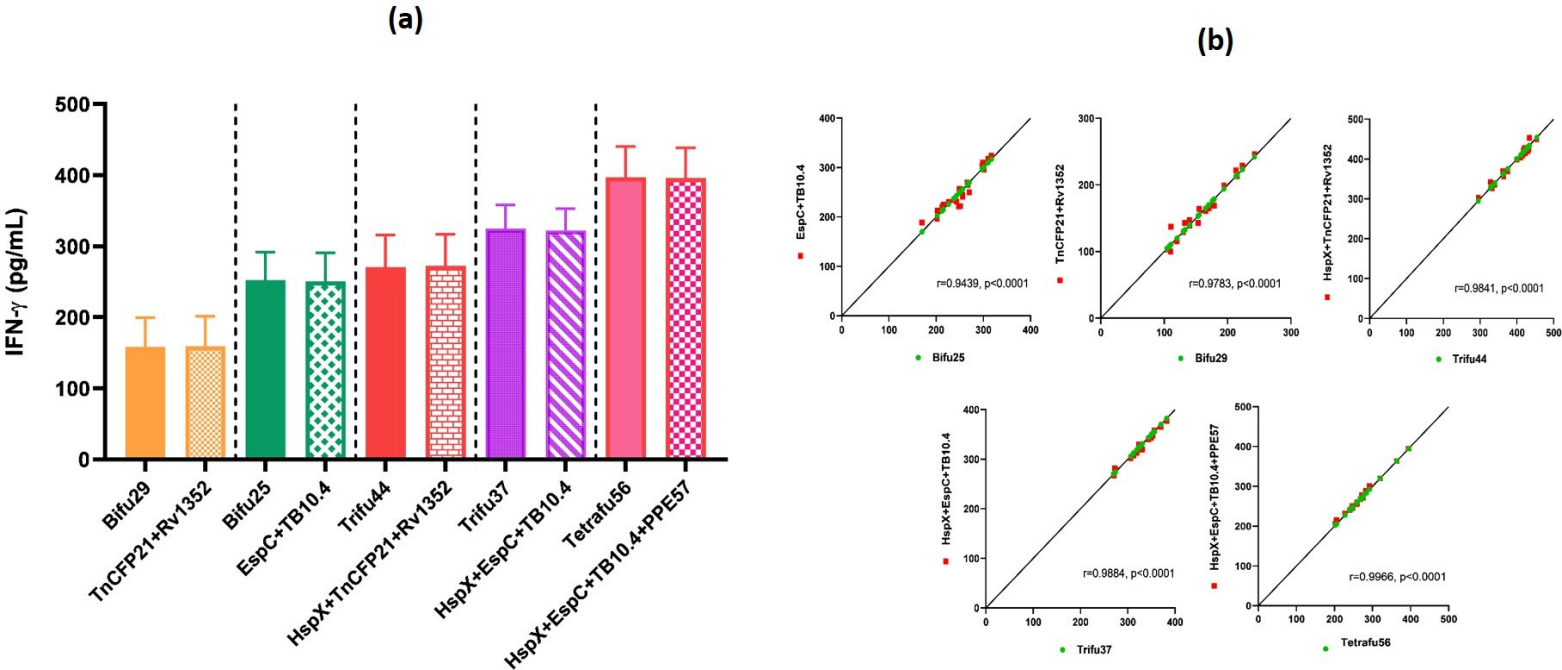
IFN-ℽ release (a) and Pearson’s correlation values (b) between IFN-ℽ released against mixtures of the antigens and their fusions. IFN-ℽ release (pg/mL) from PBMCs of active TB patients against mixtures of the antigens (red) and their fusions (green) in Pearson correlation graphs (b).

The amounts of IFN-ℽ released from PBMCs of active TB patients in response to the fusion molecules were compared with the amounts released in the presence of a mixture of equivalent amounts of the constituent antigens. The mean amounts of IFN-ℽ released for the mixtures EspC+TB10.4, TnCFP21+Rv1352, HspX+TnCFP21+Rv1352, HspX+EspC+TB10.4 and HspX+EspC+TB10.4+PPE57, were 252.1, 159.2, 270.4, 325.1 and 397.0 pg/mL, respectively. Thus the releases of IFN-ℽ from PBMCs of TB patients by the fusion molecules were almost the same as those released from the mixture of the constituent antigens in the free state. The comparative values for the amounts of IFN-ℽ released from PBMCs of TB patients are shown in Fig 5A.

The levels of IFN-ℽ release in response to the fusion antigens and their cocktails were analyzed by Pearson’s correlation coefficient (r). The correlation coefficient ranges from −1 to +1. The r value close to +1 shows that most of the data points lie on a line) of identity, which implies similarity between IFN-γ release of fusion molecules and cocktail of the component antigens. The fusion protein bifu25 and its cocktail EspC+TB10.4 showed Pearson’s r values of 0.9439 and P< 0.0001 whereas bifu29 and TnCFP21+Rv1352 showed r values of 0.9783 and P<0.0001. Trifu37 and HspX+EspC+TB10.4 showed r values of 0.9884 and P<0.0001 while trifu44 and HspX+TnCFP21+Rv1352 showed r values of 0.9841 and P<0.0001 (Fig 5B).

The similar levels of IFN-γ release against tetrafu56 and corresponding cocktail HspX+EspC+TB10.4+PPE57 with highest r value of 0.9966 and P<0.0001 showed that T-cell epitopes of the contributing proteins in tetrafu56 fusion molecule are mostly available for interaction with T-cell receptors involved in cellular activation.

### 3.4 Statistical validation of data

Induction of IFN-γ release by PBMCs of the TB patients and the healthy subjects in response to the antigens and their fusions were compared using Mann-Whitney U test. The results were considered statistically significant for **P** <0.0001. The mean, median and 95% confidence intervals for all the antigens and their fusion are shown in Table 3. The PBMCs from TB patients showed a higher IFN-γ release than that from the cells of the healthy individuals. TB patients showed the mean IFN-γ release of 100.3, 92.1, 121.4, 75.4, 75.8, 76.0 and 86.7 pg/mL in response to the single antigens EspC, HspX, TB10.4, PPE57, CFP21, TnCFP21 and Rv1352, respectively. After stimulation with the fusion antigens bifu25, bifu29, trifu37, trifu44 and tetrafu56, TB patients showed the higher mean IFN-γ release as given in Table 3.

## 4. Discussion

Protective immunity against intracellular microbes like *Mtb*, is based on the generation of CD4^+^ Th1-type CMI responses involving the release of different cytokines such as IFN-γ, TNF-α and IL-12 [2]. Activation of CD4^+^ T-cells to induce antigen specific CMI responses depends on the recognition of T cell receptors to immunogenic T cell epitopes as complexes with MHC class II molecules [43]. Thus, assessment of the release of cytokines like IFN-γ by T-cells after antigen stimulation is one strategy to validate the antigens as possible vaccine candidates. The genes encoding the MHC class II are highly polymorphic. In humans, the MHC class II locus encodes for HLA-DR, HLA-DQ and HLA-DP protein molecules to stimulate the CD4^+^ T- cell immune responses [44].

In this study, prediction of Th1-cell epitopes of Rv1352, EspC and PPE57 antigens based on their binding affinity to MHC class II molecules was determined for the first time. All peptides were predicted for their binding affinity to HLA–DR, DP and DQ supertypes. The half maximal inhibitory concentration (IC_50_) represents the binding affinity of antigenic peptides. The strongly binding peptides showing IC_50_ <50nM values were selected as potential epitopes for T-cell recognition for subsequent stimulation of the CMI system. Two epitopes from each of the antigens EspC, PPE57 and Rv1352 were recognized as potential T-cell binders. The location of the Th1 cell binding regions for EspC were 19–38 and 54–93 amino acid residues. The two binding regions predicted for PPE57 consisted of the residues 60–97 and 115–142, while for Rv1352 these were 18–35 and 84–99. Propred analysis was performed for locating the promiscuous peptides showing a binding probability of >50% of the 51 HLA-DR alleles. Based on this criteria, one peptide in EspC (residues 80–88) and two in PPE57 (residues 72–79 and 86–94) were predicted as HLA-promiscuous peptides.

Due to variable protection and low efficacy conferred by BCG against adult pulmonary TB, a multistage fusion vaccine consisting of multiple antigens from replicating and dormant stages of *Mtb* can induce strong immune responses and ensure effective protection against TB in genetically heterogenous human populations. Recombinant fusion protein-based vaccines are likely to show more safety, specificity and easy production in comparison to live attenuated vaccines. In the present study, we constructed five fusion proteins bifu25, bifu29, trifu37, trifu44 and tetrafu56 on the basis of six immunogenic single antigens from different growth phases of *Mtb*, such as EspC, HspX, TB10.4, PPE57, CFP21 and Rv1352 and analyzed their potential to induce T-cell responses for designing multistage fusion vaccines. The antigens were selected on the basis of known information regarding the presence of T-cell epitopes, bacterial growth stage and overall size of the resultant fusion construct, as size of the molecule to be expressed within a limit is important for expression in *E*. *coli*. EspC, TB10.4, PPE57, CFP21 and Rv1352 are expressed in replicating growth phase of *Mtb* while HspX is a dormancy-related protein expressed from non-replicating mycobacteria. All the six recombinant single antigens and their fusion molecules were expressed in *E*. *coli* BL21-CodonPlus (DE3)-RIPL with different expression levels. HspX is a small heat shock protein and acts as a molecular chaperone. Linking this antigen to the N-termini of bifu25 and bifu29 resulted in their soluble expression. We have reported the role of HspX as a solubilizing ligand in previous studies [45]. The purified antigenic molecules were not tested for the endotoxin content before the immunological study. However, the purified antigens shall be treated, if required, to ensure endotoxin level within the acceptable limit, before undertaking trials in the animal model, as we did in another recent study [46].

The release of IFN-γ from PBMCs of the healthy subjects (n = 10) and active pulmonary TB patients (n = 21) in response to the single and the fusion antigens was evaluated using each antigen at a concentration of 2.5 μM/well to ensure uniformity in molecular concentration. In general, the level of IFN-γ released from PBMCs of TB patients in response to the fusion molecules were not less than the total of IFN-γ released separately from each of the constituent antigens. Thus release of 252 pg/mL in the case of bifu25 as compared to the combined value of 221pg/mL for its constituent antigens. Similarly, IFN-γ release of 325 and 397 pg/mL for trifu37 and tetrafu56, respectively, edged over the total of 313 and 388 pg/mL obtained for their constituent antigens separately. Thus the T-cell epitopes of the component antigens in the fusion molecules were not only optimally available to the receptors for activation of T cells, but in the fusion form component antigen epitopes seem to exert a slight synergistic effect in stimulating the T-cells. 3D structural and CPORT analyses also revealed that multiple Th1-cell epitopes of the component antigens in trifu37 and tetrafu56 fusion molecules were arranged in a favorable orientation to interact with T-cell receptors. In the case of trifu44 the epitopes were predominantly predicted non-supporting for the interaction, but any portion of these reported to be active or supporting were enough to elicit a good response. However, PBMCs from healthy subjects in response to the individual as well as the various fusion constructs affected releases of much lower levels of IFN-γ. Release of IFN-γ from healthy PBMCs was only 26 pg/mL in response to tetrafu56, as compared to 397 pg/mL in the case of TB patients. Thus a previous exposure of PBMCs to *Mtb* antigens in plasma samples of TB patients resulted in 15-fold increase in response to tetrafu56 fusion antigen as compared to that in case of PBMCs in healthy individuals.

The similar levels of interferon gamma release in response to the fusion antigens and their cocktails were analyzed by Pearson’s correlation (Fig 5B). All the five fusion proteins and the cocktails of component antigens showed Pearson r values close to 1 ensuring the similar levels of IFN-γ release. Tetrafu56 and its cocktail HspX+EspC+TB10.4+PPE57 showed the highest r value of 0.9966 and P value of <0.0001 (Fig 5B). T-cell mediated immune response from human PBMCs against *Mtb* single and the fusion proteins showed variable tendency of IFN-γ secretion. The differences in CMI responses among the fusion proteins might reflect that some antigens contain more immunodominant epitopes that could induce higher immune responses. A high level of IFN-γ release induced by tetrafu56 fusion protein containing multiple antigens from both the replicating and the dormant stages of *Mtb*, showed its potential as a promising construct for developing a multistage subunit vaccine. These findings can be substantiated further through evaluation of the cytokines other than IFN-γ such as TNF-α and IL-2.

Several vaccine candidates are in different stages of clinical trials in different regions around the globe. However, still the ultimate goals of a wide acceptability for application in the light of the infection stage and the population specific responses is still to be achieved. Not only the issue of acceptable level of efficacy, characterization of the vaccine as a prophylactic or immunotherapeutic application need to be clearly defined.

In our study, after evaluation of the fusion molecules for the release of IFNℽ from PBMCs, the next steps to be undertaken are their validation through flow cytometry and *in-vivo* studies in animal models [46]. The fusion molecules showing promising immunogenic results on the basis of responses in PBMCs shall be validated through *in-vivo* studies.

## Conclusion

In this study Th1-cell epitopes for EspC, Rv1352 and PPE57 antigens were predicted for the first time. The molecule tetrafu56, which was constructed by fusing four different *Mtb* antigens belonging to the replicating as well as the dormant stages, generated similar level of T-cell response in releasing IFN-γ from PBMCs as compared to the total of the responses from the constituent antigens obtained separately. The fusion tetrafu56 induced 15-fold higher IFN-γ release from PBMCs of TB patients than the healthy subjects. This fusion construct seems to be a promising molecule for developing a multistage subunit vaccine.

## Supporting information

S1 Raw images (PDF)

## References

[1] Organization WH, (2020). Global tuberculosis report 2020. Geneva: WHO; 2020.

[2] Derrick SC, Yabe IM, Yang A, Morris SL. Vaccine-induced anti-tuberculosis protective immunity in mice correlates with the magnitude and quality of multifunctional CD4 T cells. Vaccine. 2011;29(16):2902–9. doi: 10.1016/j.vaccine.2011.02.010 . 21338678

[3] Chackerian AA, Perera TV, Behar SM. Gamma Interferon-Producing CD4+ T Lymphocytes in the Lung Correlate with Resistance to Infection with Mycobacterium tuberculosis. Infect immun. 2001;69(4):2666–74. doi: 10.1128/IAI.69.4.2666-2674.2001 . 11254633 PMC98205

[4] Fine PE. Variation in protection by BCG: implications of and for heterologous immunity. Lancet. 1995;346(8986):1339–45. doi: 10.1016/s0140-6736(95)92348-9 . 7475776

[5] Brandt L, Skeiky YA, Alderson MR, Lobet Y, Dalemans W, Turner OC, et al. The protective effect of the Mycobacterium bovis BCG vaccine is increased by coadministration with the Mycobacterium tuberculosis 72-kilodalton fusion polyprotein Mtb72F in M. tuberculosis-infected guinea pigs. Infect immun. 2004; 72(11):6622. doi: 10.1128/IAI.72.11.6622-6632.2004 . 15501795 PMC523007

[6] Moyle PM, Toth I. Modern subunit vaccines: development, components, and research opportunities. ChemMedChem. 2013;8(3):360–76. doi: 10.1002/cmdc.201200487 . 23316023

[7] Soleimanpour S, Yaghoubi A, Seddighinia, FS, Rezaee SR. A century of attempts to develop an effective tuberculosis vaccine: Why they failed? Int. Immunol, 2022; 109: 108791. doi: 10.1016/j.intimp.2022.108791 35487086

[8] Hatherill M, White RG, Hawn TR. Clinical Development of New TB Vaccines: Recent Advances and Next Steps. Front Microbiol. 2020; 10:3154. doi: 10.3389/fmicb.2019.03154 32082273 PMC7002896

[9] Nemes E, Hesseling AC, Tameris M, Mauff K, Downing K, Mulenga H, et al. Safety and Immunogenicity of Newborn MVA85A Vaccination and Selective, Delayed Bacille Calmette-Guerin for Infants of Human Immunodeficiency Virus- Infected Mothers: A Phase 2 Randomized, Controlled Trial. Clin. Infect. Dis.; Off. Publ. Infect. Dis. Soc. Am. 2018; 66 (4): 554–563. doi: 10.1093/cid/cix834 29028973 PMC5849090

[10] NCT03806686 CgI. Phase 2a Clinical Trial of ID93+GLA-SE Vaccine in BCGvaccinated Healthy Healthcare Workers. Quratis Inc. 2019.

[11] Daleke MH, Ummels R, Bawono P, Heringa J, Vandenbroucke-Grauls CM, Luirink J, et al. General secretion signal for the mycobacterial type VII secretion pathway. Proc Natl Acad Sci USA. 2012;109(28):11342–7. doi: 10.1073/pnas.1119453109 . 22733768 PMC3396530

[12] Li J, Shen J, Lao S, Li X, Liu J, Wu C. Mycobacterium tuberculosis Rv3615c is a highly immunodominant antigen and specifically induces potent Th1-type immune responses in tuberculosis pleurisy. Clin Sci. 2017;131(15):1859–76. doi: 10.1042/CS20170205 . 28588103

[13] Millington KA, Fortune SM, Low J, Garces A, Hingley-Wilson SM, Wickremasinghe M, et al. Rv3615c is a highly immunodominant RD1 (Region of Difference 1)-dependent secreted antigen specific for Mycobacterium tuberculosis infection. Proc Natl Acad Sci USA. 2011;108(14):5730–5. doi: 10.1073/pnas.1015153108 . 21427227 PMC3078386

[14] Kong H, Dong C, Xiong S. A novel vaccine p846 encoding Rv3615c, Mtb10. 4, and Rv2660c elicits robust immune response and alleviates lung injury induced by Mycobacterium infection. Hum Vaccin Immunother. 2014;10(2):378–90. doi: 10.4161/hv.27121 . 24280763 PMC4185893

[15] Wang J, Qie Y, Zhang H, Zhu B, Xu Y, Liu W, et al. PPE protein (Rv3425) from DNA segment RD11 of Mycobacterium tuberculosis: a novel immunodominant antigen of Mycobacterium tuberculosis induces humoral and cellular immune responses in mice. Microbiol immunol. 2008;52(4):224–30. doi: 10.1111/j.1348-0421.2008.00029.x . 18426397

[16] Zhang H, Wang J, Lei J, Zhang M, Yang Y, Chen Y, et al. PPE protein (Rv3425) from DNA segment RD11 of Mycobacterium tuberculosis: a potential B-cell antigen used for serological diagnosis to distinguish vaccinated controls from tuberculosis patients. Clin Microbiol Infect. 2007;13(2):139–45. doi: 10.1111/j.1469-0691.2006.01561.x . 17328725

[17] Ling Wang J, qing Qie Y, dong Zhu B, mei Zhang H, Xu Y, zhong Wang Q, et al. Evaluation of a recombinant BCG expressing antigen Ag85B and PPE protein Rv3425 from DNA segment RD11 of Mycobacterium tuberculosis in C57BL/6 mice. Med microbiol immunol. 2009; 198(1): 5–11. doi: 10.1007/s00430-008-0098-x . 18491134

[18] Siegrist MS, Unnikrishnan M, McConnell MJ, Borowsky M, Cheng TY, Siddiqi N, et al. Mycobacterial Esx-3 is required for mycobactin-mediated iron acquisition. Proc Natl Acad Sci USA. 2009;106(44):18792–7. doi: 10.1073/pnas.0900589106 . 19846780 PMC2774023

[19] Skjot RL, Brock I, Arend SM, Munk ME, Theisen M, Ottenhoff TH, et al. Epitope mapping of the immunodominant antigen TB10. 4 and the two homologous proteins TB10. 3 and TB12. 9, which constitute a subfamily of the esat-6 gene family. Infect immun. 2002; 70(10): 5446–53. doi: 10.1128/IAI.70.10.5446-5453.2002 . 12228269 PMC128304

[20] Davila J, McNamara LA, Yang Z. Comparison of the predicted population coverage of tuberculosis vaccine candidates Ag85B-ESAT-6, Ag85B-TB10. 4, and Mtb72f via a bioinformatics approach. PLoS One. 2012;7(7): e40882. doi: 10.1371/journal.pone.0040882 . 22815851 PMC3398899

[21] Hoang T, Aagaard C, Dietrich J, Cassidy JP, Dolganov G, Schoolnik GK, et al. ESAT-6 (EsxA) and TB10. 4 (EsxH) based vaccines for pre-and post-exposure tuberculosis vaccination. PLoS One. 2013;8(12): e80579. doi: 10.1371/journal.pone.0080579 . 24349004 PMC3861245

[22] Niu H, Hu L, Li Q, Da Z, Wang B, Tang K, et al. Construction and evaluation of a multistage *Mtb* subunit vaccine candidate Mtb10. 4–HspX. Vaccine. 2011;29(51): 9451–8. doi: 10.1016/j.vaccine.2011.10.032 . 22024175

[23] Xin Q, Niu H, Li Z, Zhang G, Hu L, Wang B, et al. Subunit vaccine consisting of multi-stage antigens has high protective efficacy against Mtb infection in mice. PLoS One. 2013;8(8): e72745. doi: 10.1371/journal.pone.0072745 . 23967337 PMC3744459

[24] Kennaway CK, Benesch JL, Gohlke U, Wang L, Robinson CV, Orlova EV, et al. Dodecameric structure of the small heat shock protein Acr1 from Mycobacterium tuberculosis. J Biol Chem. 2005;280(39):33419–25. doi: 10.1074/jbc.M504263200 . 16046399

[25] Singh S, Saraav I, Sharma S. Immunogenic potential of latency associated antigens against Mycobacterium tuberculosis. Vaccine. 2014;32(6):712–6. doi: 10.1016/j.vaccine.2013.11.065 . 24300592

[26] Li Q, Yu H, Zhang Y, Wang B, Jiang W, Da Z, et al. Immunogenicity and protective efficacy of a fusion protein vaccine consisting of antigen Ag85B and HspX against Mycobacterium tuberculosis infection in mice. Scand J immunol. 2011;73(6):568–76. doi: 10.1111/j.1365-3083.2011.02531.x . 21323695

[27] Covert BA, Spencer JS, Orme IM, Belisle JT. The application of proteomics in defining the T cell antigens of Mycobacterium tuberculosis. Proteomics: Int Edt. 2001;1(4):574–86. doi: 10.1002/1615-9861(200104)1:4<574::AID-PROT574>3.0.CO;2-8 . 11681210

[28] Grover A, Ahmed MF, Verma I, Sharma P, Khuller GK. Expression and purification of the Mycobacterium tuberculosis complex-restricted antigen CFP21 to study its immunoprophylactic potential in mouse model. Protein Expr Purif. 2006;48(2):274–80. doi: 10.1016/j.pep.2006.03.010 . 16716602

[29] Vita R, Zarebski L, Greenbaum JA, Emami H, Hoof I, Salimi N, et al. The immune epitope database 2.0. Nucleic Acids Res. 2010;38(suppl_1):D854–62. doi: 10.1093/nar/gkp1004 . 19906713 PMC2808938

[30] Kim Y, Ponomarenko J, Zhu Z, Tamang D, Wang P, Greenbaum J, et al. Immune epitope database analysis resource. Nucleic Acids Res. 2012;40(W1):W525–30. doi: 10.1093/nar/gks438 . 22610854 PMC3394288

[31] Greenbaum J, Sidney J, Chung J, Brander C, Peters B, Sette A. Functional classification of class II human leukocyte antigen (HLA) molecules reveals seven different supertypes and a surprising degree of repertoire sharing across supertypes. Immunogenetics. 2011;63(6):325–35. doi: 10.1007/s00251-011-0513-0 . 21305276 PMC3626422

[32] Reynisson B, Barra C, Kaabinejadian S, Hildebrand WH, Peters B, Nielsen M. Improved prediction of MHC II antigen presentation through integration and motif deconvolution of mass spectrometry MHC eluted ligand data. J proteome Res. 2020; 19(6):2304–15. doi: 10.1021/acs.jproteome.9b00874 . 32308001

[33] Singh H, Raghava GP. ProPred: prediction of HLA-DR binding sites. Bioinformatics. 2001;17(12):1236–7. doi: 10.1093/bioinformatics/17.12.1236 . 11751237

[34] Mustafa AS. In silico binding predictions for identification of HLA-DR-promiscuous regions and epitopes of Mycobacterium tuberculosis protein MPT64 (Rv1980c) and their recognition by human Th1 cells. Med Princ Pract. 2010; 19(5): 367–72. doi: 10.1159/000316375 . 20639660

[35] Arif S, Akhter M, Khaliq A, un Nisa Z, Khan IH, Akhtar MW. Serodiagnostic evaluation of fusion proteins from multiple antigens of Mycobacterium tuberculosis for active TB. Tuberculosis. 2021;127:102053. doi: 10.1016/j.tube.2021.102053 . 33561630

[36] Akhter M, Arif S, Khaliq A, un Nisa Z, Khan IH, Akhtar MW. Designing fusion molecules from antigens of Mycobacterium tuberculosis for detection of multiple antibodies in plasma of TB patients. Tuberculosis. 2020;124:101981. doi: 10.1016/j.tube.2020.101981 . 32810724

[37] Vries de Vries SJ, Bonvin AM. CPORT: a consensus interface predictor and its performance in prediction-driven docking with HADDOCK. PLoS One. 2011;6(3): e17695. doi: 10.1371/journal.pone.0017695 . 21464987 PMC3064578

[38] Somerville W, Thibert L, Schwartzman K, Behr MA. Extraction of Mycobacterium tuberculosis DNA: a question of containment. J Clin Microbiol 2005;43(6):2996–7. doi: 10.1128/JCM.43.6.2996-2997.2005 . 15956443 PMC1151963

[39] Khurshid S, Khalid R, Afzal M, Akhtar MW. Truncation of PstS1 antigen of Mycobacterium tuberculosis improves diagnostic efficiency. Tuberculosis 2013;93 (6):654–9. doi: 10.1016/j.tube.2013.07.005 . 23978525

[40] Bradford MM. A rapid and sensitive method for the quantitation of microgram quantities of protein utilizing the principle of protein-dye binding. Anal Biochem 1976;72(1–2):248–54. doi: 10.1006/abio.1976.9999 . 942051

[41] World Health Organization. TB manual: national tuberculosis programme guidelines. Copenhagen: WHO Regional Office for Europe; 2001.

[42] Jaatinen T, Laine J. Isolation of mononuclear cells from human cord blood by Ficoll‐Paque density gradient. Curr Protoc Stem Cell Biol. 2007; 1(1): 2A–1. doi: 10.1002/9780470151808.sc02a01s1 . 18785173

[43] Al-Attiyah R, Shaban FA, Wiker HG, Oftung F, Mustafa AS. Synthetic peptides identify promiscuous human Th1 cell epitopes of the secreted mycobacterial antigen MPB70. Infect immun. 2003;71(4):1953–60. doi: 10.1128/IAI.71.4.1953-1960.2003 . 12654813 PMC152036

[44] Garstka MA, Fish A, Celie PH, Joosten RP, Janssen GM, Berlin I, et al. The first step of peptide selection in antigen presentation by MHC class I molecules. Proc Natl Acad Sci USA. 2015;112(5):1505–10. doi: 10.1073/pnas.1416543112 . 25605945 PMC4321303

[45] Khalid R, Afzal M, Khurshid S, Paracha RZ, Khan IH, Akhtar MW. Fusion molecules of heat shock protein HSPX with other antigens of Mycobacterium tuberculosis show high potential in serodiagnosis of tuberculosis. PLoS One. 2016; 11(9): e0163349. doi: 10.1371/journal.pone.0163349 . 27654048 PMC5031420

[46] Sulman S, Savidge BO, Alqaseer K, Das MK, Nezam Abadi N, Pearl JE, et al. Balance between Protection and Pathogenic Response to Aerosol Challenge with Mycobacterium tuberculosis (Mtb) in Mice Vaccinated with TriFu64, a Fusion Consisting of Three *Mtb* Antigens. Vaccines. 2021; May;9(5):519. doi: 10.3390/vaccines9050519 34070048 PMC8158147

