# Supplementary material for: Fusion peptide constructs from antigens of M. tuberculosis producing high T-cell mediated immune response
Source: PLoS One. 2022 Sep 29;17(9):e0271126. doi: 10.1371/journal.pone.0271126 (PMC9521936; doi:10.1371/journal.pone.0271126)
Supplement: S1 Raw images — (PDF) [file pone.0271126.s001.pdf]

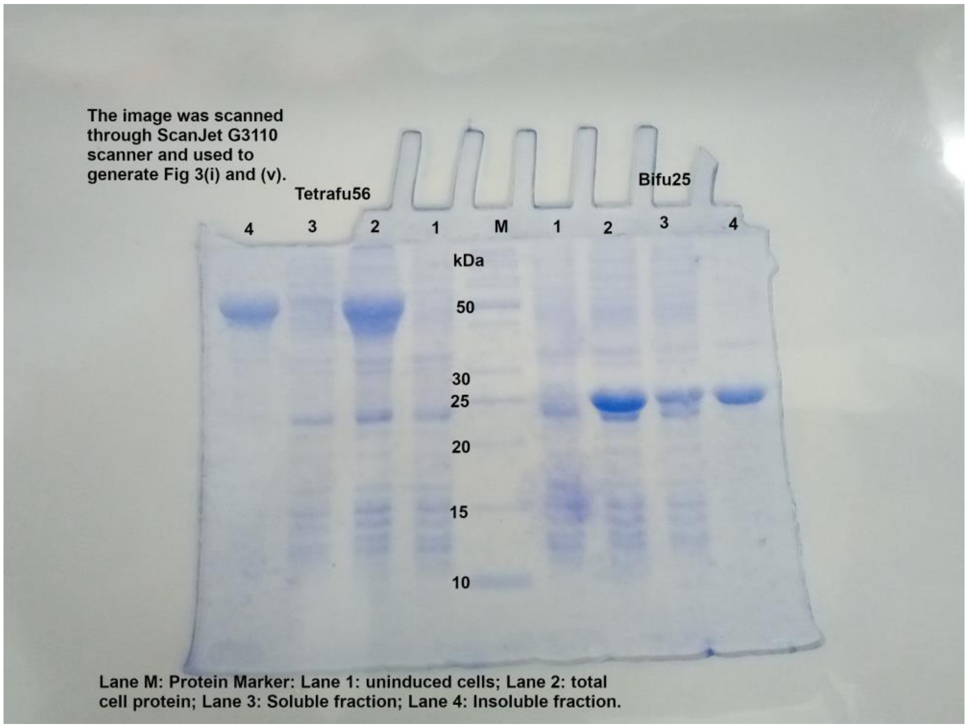
The image was scanned through ScanJet G3110 scanner and used to generate Fig 3(i) and (v).
Tetrafu56
Bifu25
4
3
2
1
M
1
2
3
4
kDa
50
30
25
20
15
10
Lane M: Protein Marker: Lane 1: uninduced cells; Lane 2: total cell protein; Lane 3: Soluble fraction; Lane 4: Insoluble fraction.

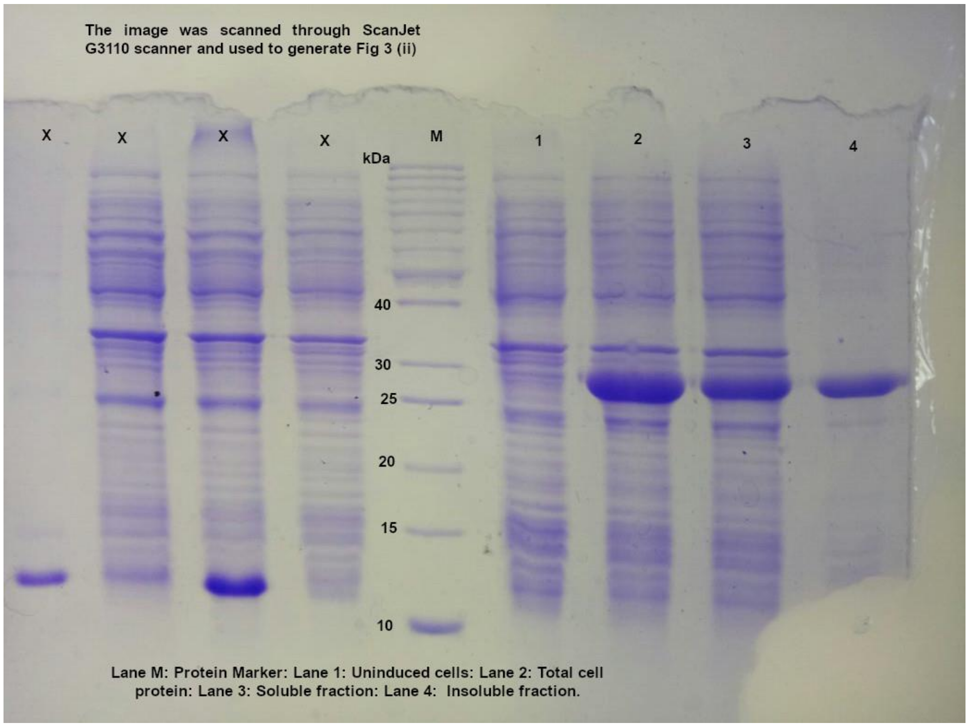
The image was scanned through ScanJet G3110 scanner and used to generate Fig 3 (ii)
X
X
X
X
M
1
2
3
4
kDa
40
30
25
20
15
10
Lane M: Protein Marker: Lane 1: Uninduced cells: Lane 2: Total cell protein: Lane 3: Soluble fraction: Lane 4: Insoluble fraction.

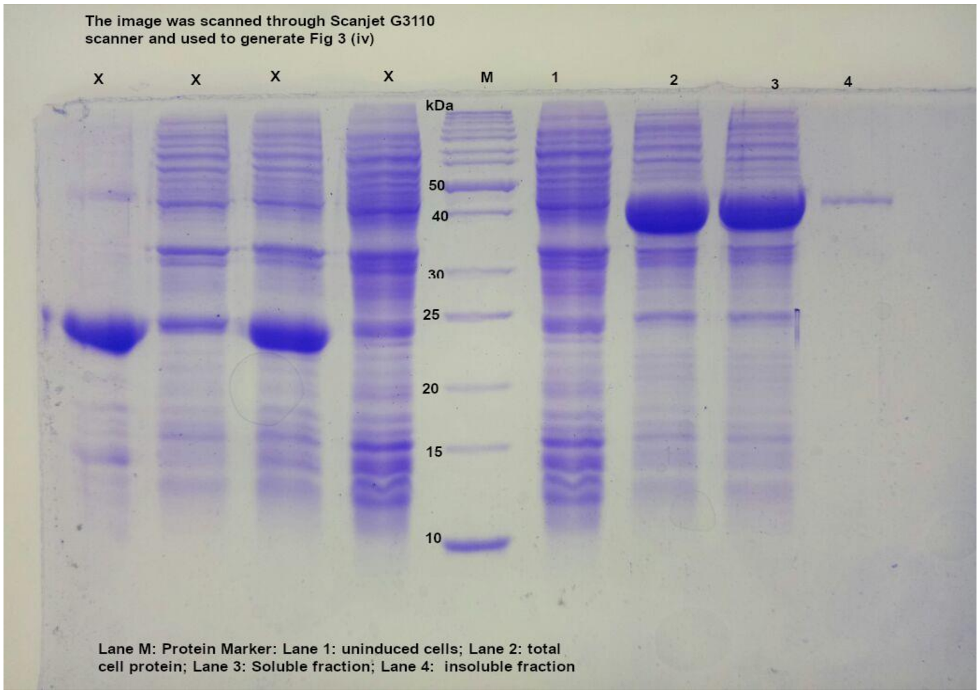
The image was scanned through Scanjet G3110
scanner and used to generate Fig 3 (iv)
X
X
X
X
M
1
2
3
4
kDa
50
40
30
25
20
15
10
Lane M: Protein Marker: Lane 1: uninduced cells; Lane 2: total
cell protein; Lane 3: Soluble fraction; Lane 4: insoluble fraction

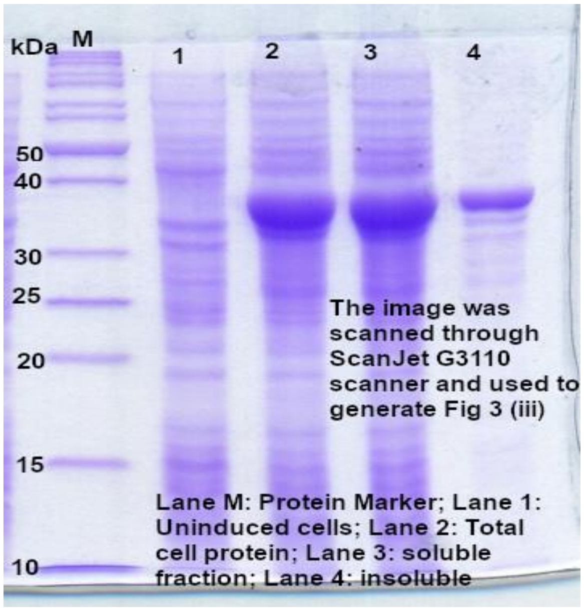
kDa
M
1
2
3
4
50
40
30
25
20
15
10
The image was
scanned through
ScanJet G3110
scanner and used to
generate Fig 3 (iii)
Lane M: Protein Marker; Lane 1:
Uninduced cells; Lane 2: Total
cell protein; Lane 3: soluble
fraction; Lane 4: insoluble
